# Supplementary material for: Resolving the full spectrum of human genome variation using Linked-Reads
Source: Genome Res. 2019 Apr;29(4):635–45. doi: 10.1101/gr.234443.118 (PMC6442396; doi:10.1101/gr.234443.118)
Supplement: Supplemental Material [file supp_gr.234443.118_Supplemental_longranger-2.2.zip › longranger-2.2.tar.bz2!/lr2.2-public/lib/stan/cmdstan/stan/doc/api/html/meta_2traits_8hpp.html]

Stan: src/stan/meta/traits.hpp File Reference


|  |  |
| --- | --- |
|  | Stan  2.5.0  probability, sampling & optimization |


- Main Page
- Related Pages
- Namespaces
- Classes
- Files

- File List
- File Members

All Classes Namespaces Files Functions Variables Typedefs Enumerations Enumerator Friends Macros Pages

- src
- stan
- meta

Classes |
Namespaces |
Functions

traits.hpp File Reference

`#include <vector>`  
`#include <boost/type_traits.hpp>`  
`#include <boost/type_traits/is_arithmetic.hpp>`  
`#include <boost/math/tools/promotion.hpp>`  
`#include <stan/agrad/fwd/fvar.hpp>`  
`#include <stan/agrad/rev/var.hpp>`  
`#include <stan/math/matrix/Eigen.hpp>`  
`#include <stan/math/matrix/meta/value_type.hpp>`  
`#include <stan/math/meta/value_type.hpp>`

Go to the source code of this file.

|  |  |
| --- | --- |
| Classes | |
| struct | stan::error\_index |
|  | |
| struct | stan::is\_constant< T > |
|  | Metaprogramming struct to detect whether a given type is constant in the mathematical sense (not the C++ `const` sense). More... |
|  | |
| struct | stan::is\_constant\_struct< T > |
|  | Metaprogram to determine if a type has a base scalar type that can be assigned to type double. More... |
|  | |
| struct | stan::is\_constant\_struct< std::vector< T > > |
|  | |
| struct | stan::is\_constant\_struct< Eigen::Matrix< T, R, C > > |
|  | |
| struct | stan::is\_constant\_struct< Eigen::Block< T > > |
|  | |
| struct | stan::is\_vector< T > |
|  | |
| struct | stan::is\_vector< const T > |
|  | |
| struct | stan::is\_vector< std::vector< T > > |
|  | |
| struct | stan::is\_vector< Eigen::Matrix< T, Eigen::Dynamic, 1 > > |
|  | |
| struct | stan::is\_vector< Eigen::Matrix< T, 1, Eigen::Dynamic > > |
|  | |
| struct | stan::is\_vector< Eigen::Block< T > > |
|  | |
| struct | stan::scalar\_type< T > |
|  | Metaprogram structure to determine the base scalar type of a template argument. More... |
|  | |
| struct | stan::size\_of\_helper< T, is\_vec > |
|  | |
| struct | stan::size\_of\_helper< T, true > |
|  | |
| struct | stan::scalar\_type< Eigen::Matrix< T, Eigen::Dynamic, Eigen::Dynamic > > |
|  | |
| struct | stan::scalar\_type< T \* > |
|  | |
| struct | stan::is\_vector\_like< T > |
|  | |
| struct | stan::is\_vector\_like< T \* > |
|  | |
| struct | stan::is\_vector\_like< const T > |
|  | |
| struct | stan::is\_vector\_like< Eigen::Matrix< T, Eigen::Dynamic, Eigen::Dynamic > > |
|  | |
| class | stan::VectorView< T, is\_array, throw\_if\_accessed > |
|  | VectorView is a template metaprogram that takes its argument and allows it to be used like a vector. More... |
|  | |
| class | stan::VectorView< const T, is\_array, throw\_if\_accessed > |
|  | VectorView that has const correctness. More... |
|  | |
| class | stan::VectorView< const double, false, false > |
|  | |
| class | stan::DoubleVectorView< used, is\_vec > |
|  | DoubleVectorView allocates double values to be used as intermediate values. More... |
|  | |
| class | stan::DoubleVectorView< true, false > |
|  | |
| class | stan::DoubleVectorView< true, true > |
|  | |
| struct | stan::return\_type< T1, T2, T3, T4, T5, T6 > |
|  | Metaprogram to calculate the base scalar return type resulting from promoting all the scalar types of the template parameters. More... |
|  | |
| struct | stan::is\_fvar< T > |
|  | |
| struct | stan::is\_fvar< stan::agrad::fvar< T > > |
|  | |
| struct | stan::is\_var< T > |
|  | |
| struct | stan::is\_var< stan::agrad::var > |
|  | |
| struct | stan::contains\_fvar< T1, T2, T3, T4, T5, T6 > |
|  | Metaprogram to calculate the base scalar return type resulting from promoting all the scalar types of the template parameters. More... |
|  | |
| struct | stan::is\_var\_or\_arithmetic< T1, T2, T3, T4, T5, T6 > |
|  | |
| struct | stan::scalar\_type\_pre< T > |
|  | Metaprogram structure to determine the type of first container of the base scalar type of a template argument. More... |
|  | |
| class | stan::VectorViewMvt< T, is\_array, throw\_if\_accessed > |
|  | |
| class | stan::VectorViewMvt< const T, is\_array, throw\_if\_accessed > |
|  | VectorViewMvt that has const correctness. More... |
|  | |

|  |  |
| --- | --- |
| Namespaces | |
|  | stan |
|  | Probability, optimization and sampling library. |
|  | |

|  |  |
| --- | --- |
| Functions | |
| template<typename T > | |
| T | stan::get (const T &x, size\_t n) |
|  | |
| template<typename T > | |
| T | stan::get (const std::vector< T > &x, size\_t n) |
|  | |
| template<typename T , int R, int C> | |
| T | stan::get (const Eigen::Matrix< T, R, C > &m, size\_t n) |
|  | |
| template<typename T > | |
| size\_t | stan::length (const T &) |
|  | |
| template<typename T > | |
| size\_t | stan::length (const std::vector< T > &x) |
|  | |
| template<typename T , int R, int C> | |
| size\_t | stan::length (const Eigen::Matrix< T, R, C > &m) |
|  | |
| template<typename T > | |
| size\_t | stan::size\_of (const T &x) |
|  | |
| template<typename T1 , typename T2 > | |
| size\_t | stan::max\_size (const T1 &x1, const T2 &x2) |
|  | |
| template<typename T1 , typename T2 , typename T3 > | |
| size\_t | stan::max\_size (const T1 &x1, const T2 &x2, const T3 &x3) |
|  | |
| template<typename T1 , typename T2 , typename T3 , typename T4 > | |
| size\_t | stan::max\_size (const T1 &x1, const T2 &x2, const T3 &x3, const T4 &x4) |
|  | |
| template<typename T > | |
| size\_t | stan::length\_mvt (const T &) |
|  | |
| template<typename T , int R, int C> | |
| size\_t | stan::length\_mvt (const Eigen::Matrix< T, R, C > &) |
|  | |
| template<typename T , int R, int C> | |
| size\_t | stan::length\_mvt (const std::vector< Eigen::Matrix< T, R, C > > &x) |
|  | |
| template<typename T1 , typename T2 > | |
| size\_t | stan::max\_size\_mvt (const T1 &x1, const T2 &x2) |
|  | |
| template<typename T1 , typename T2 , typename T3 > | |
| size\_t | stan::max\_size\_mvt (const T1 &x1, const T2 &x2, const T3 &x3) |
|  | |
| template<typename T1 , typename T2 , typename T3 , typename T4 > | |
| size\_t | stan::max\_size\_mvt (const T1 &x1, const T2 &x2, const T3 &x3, const T4 &x4) |
|  | |


---

[ Stan Home Page ]

*© 2011–2014,
Stan Development Team.*
